# Long-term outcomes of less drug-eluting stents by the use of drug-coated balloons in *de novo* coronary chronic total occlusion intervention: A multicenter observational study

**DOI:** 10.3389/fcvm.2023.1045859

**Published:** 2023-03-03

**Authors:** Xi Wang, Xinyue Yang, Wenjie Lu, Liang Pan, Zhanying Han, Sancong Pan, Yingguang Shan, Xule Wang, Xiaolin Zheng, Ran Li, Yongjian Zhu, Peng Qin, Qiangwei Shi, Shuai Zhou, Wencai Zhang, Sen Guo, Peisheng Zhang, Xiaofei Qin, Guoju Sun, Zhongsheng Qin, Zhenwen Huang, Chunguang Qiu

**Affiliations:** ^1^Department of Cardiovascular Medicine, The First Affiliated Hospital of Zhengzhou University, Zhengzhou, China; ^2^Department of Cardiovascular Medicine, Jincheng People’s Hospital, Jincheng, China; ^3^Department of Geriatric Cardiology, The First Affiliated Hospital of Zhengzhou University, Zhengzhou, China; ^4^Department of Cardiology, The Fifth Affiliated Hospital of Zhengzhou University, Zhengzhou, China

**Keywords:** coronary heart disease, drug-coated balloon, drug-eluting stent, chronic total occlusion, percutaneous coronary intervention

## Abstract

**Background:**

Data on drug-coated balloons (DCB) for *de novo* coronary chronic total occlusion (CTO) are limited. We aimed to investigate the long-term outcomes of substitution of drug-eluting stents (DES) by DCB.

**Methods:**

We compared the outcomes of less DES strategy (DCB alone or combined with DES) and DES-only strategy in treating *de novo* coronary CTO in this prospective, observational, multicenter study. The primary endpoints were major adverse cardiovascular events (MACE), target vessel revascularization, myocardial infarction, and death during 3-year follow-up. The secondary endpoints were late lumen loss (LLL) and restenosis until 1-year after operation.

**Results:**

Of the 591 eligible patients consecutively enrolled between January 2015 and December 2019, 281 (290 lesions) were treated with DCB (DCB-only or combined with DES) and 310 (319 lesions) with DES only. In the DCB group, 147 (50.7%) lesions were treated using DCB-only, and the bailout stenting rate was relatively low (3.1%). The average stent length per lesion in the DCB group was significantly shorter compared with the DES-only group (21.5 ± 25.5 mm vs. 54.5 ± 26.0 mm, *p* < 0.001). A total of 112 patients in the DCB group and 71 patients in the DES-only group (38.6% vs. 22.3%, *p* < 0.001) completed angiographic follow-up until 1-year, and LLL was much less in the DCB group (−0.08 ± 0.65 mm vs. 0.35 ± 0.62 mm, *p* < 0.001). There were no significant differences in restenosis occurrence between the two groups (20.5% vs. 19.7%, *p* > 0.999). The Kaplan–Meier estimates of MACE at 3-year (11.8% vs. 12.0%, log-rank *p* = 0.688) was similar between the groups.

**Conclusion:**

Percutaneous coronary intervention with DCB is a potential “stent-less” therapy for *de novo* CTO lesions with satisfactory long-term clinical results compared to the DES-only approach.

## 1. Introduction

Coronary chronic total occlusion (CTO) is characterized by thrombolysis in myocardial infarction (TIMI) grade 0, for at least 3 months, determined angiographically or clinically. CTO is found in 15–25% of individuals undergoing coronary angiography (1, 2). Successful CTO revascularization can lead to symptomatic angina relief, and improved cardiac function and clinical outcomes (3, 4). Recently, CTO percutaneous coronary intervention (PCI) has advanced rapidly owing to the growing experience of interventional cardiologists in antithrombotic drug management, expanding application of drug-eluting stents (DES), and procedural strategies (5). Currently, new-generation DES is the most widely used intervention in recanalized CTO and reduces the incidence of restenosis and major adverse cardiovascular events (MACE) (5, 6). However, some disadvantages remain, including risks of restenosis and stent thrombosis (ST) due to long stent implantation, unsuitability for stenting immediately after predilation, and distal vessel remodeling after antegrade flow restoration (7, 8).

Drug-coated balloons (DCB) were first reported by Scheller to treat in-stent restenosis (ISR) (9). Compared with DES, DCB can rapidly deliver anti-proliferative drugs to the coronary vessel wall, and no permanent metal implants are left after PCI. Owing to the potential advantages of reducing adverse events associated with DES and shortening the period of dual antiplatelet therapy (DAPT), DCB angioplasty is becoming a popular alternative treatment for coronary lesions. Previous studies have demonstrated that DCB has sound clinical effects and safety in bare metal stent (BMS)/DES ISR (10).

In terms of small vessel disease, DCB is also demonstrably safe and effective, and increasing evidence supports DCB in treating acute myocardial infarction (MI), bifurcation lesions, and large vessel disease (11). However, only a few studies with a small sample size and short follow-up duration have evaluated DCB in treating *de novo* CTO lesions (12–14). We aimed to compare the efficacy and safety of less DES strategy (DCB-only or combined with DES) and DES-only strategy in treating *de novo* CTO lesions in a prospective, multicenter, observational study with long-term follow-up.

## 2. Methods

### 2.1. Patient population

**Figure 1:**
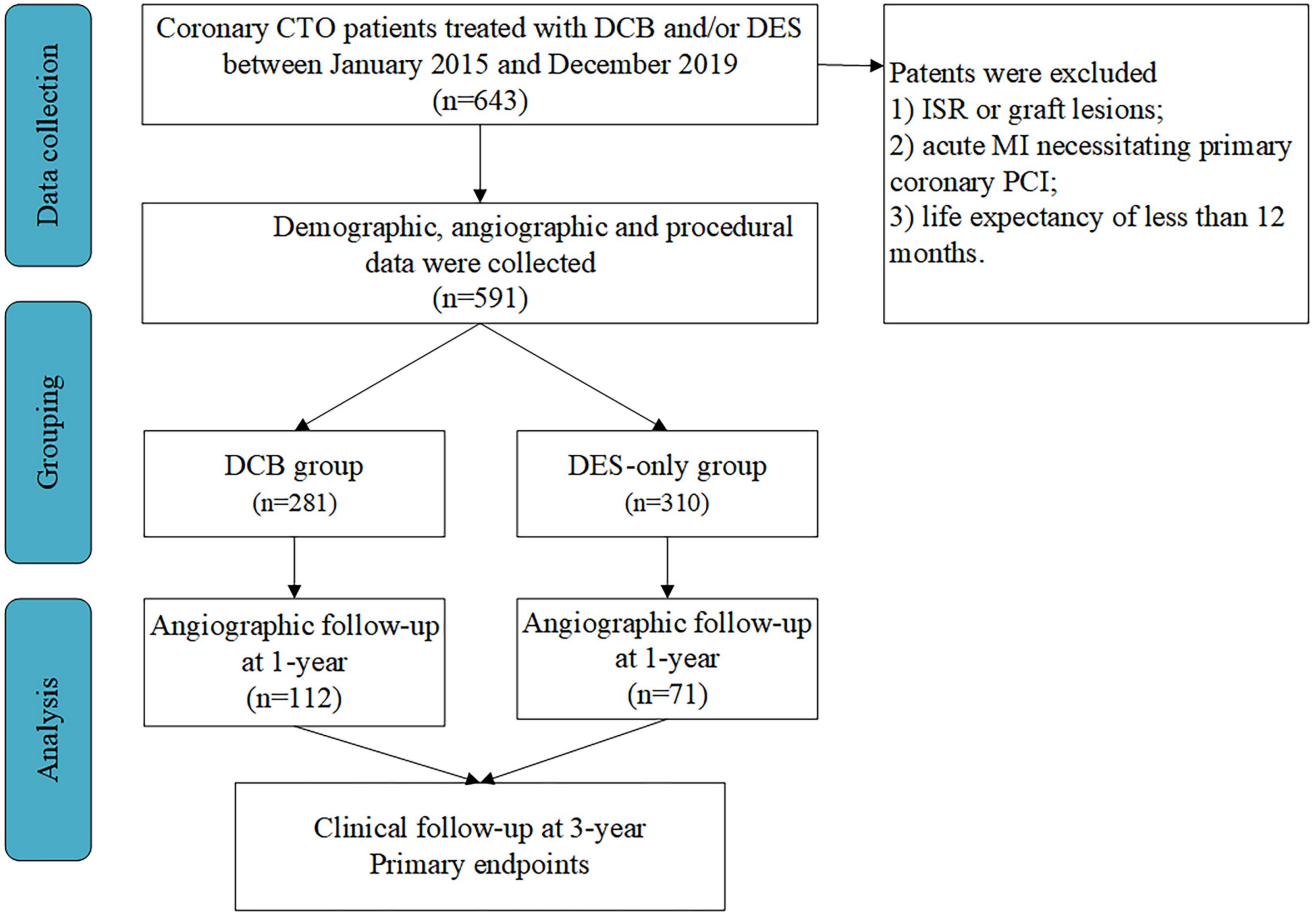
Study population. CTO, chronic total occlusion; DCB, drug-coated balloon; DES, drug-eluting stent; ISR, in-stent restenosis; MI, myocardial infarction; PCI, percutaneous coronary intervention.

This prospective observational study was performed in three high-volume PCI centers in China (15, 16). Patients with coronary CTO treated with DCB and/or DES between January 2015 and December 2019 were enrolled consecutively. Inclusion criteria were (1) coronary CTO lesions, defined as TIMI grade 0 with a minimum period of 3 months, as demonstrated angiographically or clinically, and (2) recanalized CTO with DCB angioplasty and/or DES implantation. Exclusion criteria were (1) ISR or graft lesions; (2) acute ST-segment elevation MI necessitating primary PCI, and (3) life expectancy of less than 12 months (Figure 1).

This research was performed according to the Declaration of Helsinki. The local ethics committee approved the research and all patients signed written informed consent forms. This research was not sponsored by any external source.

### 2.2. PCI procedure

PCI was performed according to standard procedures. All patients were administered aspirin and clopidogrel or ticagrelor. Heparin was routinely administered during the procedure at a loading dose of 80–100 IU/kg followed by 1,000 IU per hour. Baseline angiography was completed with at least two near-orthogonal images to show the target lesion following an intracoronary injection of nitroglycerin (100–200 μg). Recanalization strategies for CTO lesions were decided by interventionists.

In the DCB group, predilation was performed with either semi-compliant balloons or noncompliant, cutting, scoring, or dual wire balloons. If predilation was satisfactory, DCB was used to cover the entire target or partial lesion in cases of dissection < type C and residual stenosis ≤50%. New-generation DES implantation was performed as part of an initial hybrid strategy combining DCB and DES, or as bailout stents in segments with flow-limiting dissection or significant residual stenosis (> 50%) after DCB angioplasty. Paclitaxel-coated balloons (SeQuent ™ Please, B. Braun, Melsungen, Germany) were used in all patients undergoing DCB angioplasty. New-generation DES was used in patients treated with DES only. Four types of DES were implanted: Resolute IntegrityTM (Medtronic, Santa Rosa, CA, USA), SynergyTM (Boston Scientific, Maple Grove, MN, USA), Excrossal (JW Medical System, China), Excel (JW Medical System, China).

Administration of post-procedure glycoprotein IIb/IIIa receptor inhibitors was based on the patient’s condition. Patients without DES implantation received DAPT for at least 1 month, and DAPT was maintained for 6–12 months in patients after stenting.

### 2.3. Angiographic analysis

According to the results of CAG, the calcification lesions were classified as grade 0 (no calcification). Grade I (mild calcification) can only see the faint and fuzzy high-density shadow when the heart is beating, but cannot see the calcification shadow completely when the heart is not beating. Grade II (moderate calcification) sees sharper, easier-to-see dense shadows when the heart beats. Grade III (severe calcification) can be seen with a clear, dense shadow in both beating and non-beating hearts (17). Edge detection methods (QAngio XA 7.3 version; Medis Medical Imaging, Leiden, Netherlands) were used to provide quantitative coronary angiography (QCA) measurements during intervention and follow-up angiography. Lesion length, reference vessel diameter (RVD), minimum luminal diameter (MLD), and diameter stenosis percentage were measured. The difference in MLD immediately after intervention and during follow-up angiography was used to compute late lumen loss (LLL).

### 2.4. Clinical endpoints and definitions

The primary endpoints were MACE, defined as all-cause death, MI, and target vessel revascularization (TVR). The secondary end points were LLL and restenosis. Periprocedural serious adverse events included ST, MI, and death during hospitalization. According to the QCA results, ISR was defined as a restenosis of 50% within the DCB or DES area or at its adjacent 5-mm segments. Death was considered cardiogenic unless there is an apparent non-cardiogenic cause. The Fourth Universal Definition of Myocardial Infarction defined procedure-related, nonprocedural, or after-discharge MI (18). Revascularization of the target lesion (lesion within 5 mm from the edge of each end) and target vessel was defined as target lesion revascularization (TLR) and TVR, respectively. The criteria provided by the Academic Research Consortium were used to classify the ST (19).

### 2.5. Follow-up

Follow-up was achieved for a total of 3 years using clinical visits or phone calls every 3 months. Angiograms were performed 6 and 12 months after PCI or when clinically necessary, but this was not mandatory.

### 2.6. Statistical analysis

Data were presented as the mean ± standard deviation or median (interquartile range). The *t*-test or non-parametric test was used to assess differences between continuous variables, and the chi-square test or Fisher’s exact test was used for categorical variables. The cumulative incidence of outcomes was estimated from the Kaplan–Meier curve and compared using the log-rank test. The multivariate Cox proportional regression model was used to adjust for potential confounders, including baseline patient characteristics, target vessels, calcification, lesion length, MLD after PCI, and RVD. Statistical analysis was conducted using SPSS 23.0 (IBM SPSS, SPSS Inc.). A two-sided *p* value of <0.05 was considered statistically significant.

## 3. Results

### 3.1. Population

We identified 591 consecutive eligible patients with CTO and 281 patients (47.5%) received DCB between January 2015 and December 2019. The mean age was 58.80 ± 10.87 years, and 72.93% of the patients were male. A high percentage of patients had hypertension (55.16%), diabetes (35.36%), and hypercholesterolemia (57.19%). Majority (77.9%) of the patients presented with multivessel diseases. A total of 281 patients treated with DCB (DCB-only or DCB combined with DES for sequential segments of a lesion) and 310 patients with DES-only implantations were enrolled. There were no significant differences in the baseline patient characteristics between the groups (Table 1).

**Table 1:** Baseline patient characteristics.

	DCB (*n* = 281)	DES-only (*n* = 310)	*P*
Age, years	58.4 ± 10.9	59.1 ± 10.8	0.431
Male	207 (73.7%)	224 (72.3%)	0.712
Comorbidities			
Hypertension	154 (54.8%)	172 (55.5%)	0.869
Diabetes mellitus	105 (37.4%)	104 (33.6%)	0.344
Hypercholesterolemia	150 (53.4%)	188 (60.7)	0.081
Family history of CAD	63 (22.4%)	58 (18.71%)	0.307
Previous MI	76 (27.1%)	90 (29.0%)	0.647
Previous PCI	39 (13.9%)	41 (13.2%)	0.904
Previous CABG	4 (1.4%)	10 (3.2%)	0.182
Current/ex-smoker	110 (39.2%)	122 (39.4%)	>0.999
Unstable angina	65 (23.1%)	69 (22.3%)	0.844
Number of diseased vessels			
1	62 (22.1%)	53 (17.1%)	0.204
2	97 (34.5%)	103 (33.2%)	
3	122 (43.4%)	154 (49.7%)	
Multivessel disease	202 (71.9%)	213 (68.7%)	0.419
Complete revascularization	210 (74.7%)	226 (72.9%)	0.640
Ejection fraction, %	58.2 ± 7.0	57.0 ± 8.0	0.060
Medication during hospitalization			
Aspirin	281 (100.0%)	310 (100.0%)	>0.999
Clopidogrel	99 (35.2%)	101 (32.6%)	0.542
ticagrelor	182 (64.8%)	209 (67.4%)	0.542
statin	281 (100.0%)	310 (100.0%)	>0.999
Beta-blocker	194 (69.0%)	221 (71.3%)	0.589

Values are mean ± SD or *n* (%). CABG, coronary artery bypass graft surgery; CAD, coronary artery disease; DCB, drug-coated balloon; DES, drug-eluting stent; MI, myocardial infarction; PCI, percutaneous coronary intervention.

### 3.2. Procedural characteristics

The most common PCI access was the transradial artery (93.1% vs. 91.2%, *p* = 0.452) in both groups. The most frequent target vessels were the left anterior descending (LAD) (39.7%) and right coronary artery (RCA) (40.0%) in the DCB group and the RCA (49.5%) in the DES-only group (*p* = 0.031). The percentage of calcified lesions was relatively higher in the DES-only group (7.6% vs. 11.9%, *p* = 0.078). Noncompliant balloons, cutting balloons, and nonslip element balloons were more frequently used in the DCB group. The incidence of dissection after post-dilation was higher in the DES-only group than that in the DCB group (No dissection: 30.1% vs. 40.0%; A-B dissection: 53.9% vs. 50.7%; C-F dissection: 16.0% vs. 9.3%, *p* = 0.007). The incidence of type A-B and type C-F dissection after DCB angioplasty in the DCB group was 49.7 and 12.1%, and there was no dissection after stent implantation in the DES-only group (*p* < 0.001). Nine lesions (3.1%) in the DCB group required bailout stents. Table 2 summarizes the procedure and lesion characteristics.

**Table 2:** Characteristics of procedures and lesions.

	DCB (*n* = 290)	DES-only (*n* = 319)	*P*
Access			
Trans-radial	270 (93.1%)	291 (91.2%)	0.452
Trans-femoral	20 (6.9%)	28 (8.8%)	
Target vessel			
LAD	115 (39.7%)	116 (36.4%)	0.031
LCX	59 (20.3%)	45 (14.1%)	
RCA	116 (40.0%)	158 (49.5%)	
Pre-dilation			
Semi-compliant balloon (%)	277 (95.5%)	311 (97.5%)	0.191
Non-compliant balloon (%)	45 (15.5%)	17 (5.3%)	<0.001
Cutting balloon (%)	56 (19.3%)	33 (10.3%)	0.002
NSE balloon (%)	79 (27.2%)	24 (7.5%)	<0.001
Dual wire balloon (%)	13 (4.5%)	6 (1.9%)	0.100
Moderate/severe Calcification	22 (7.6%)	38 (11.9%)	0.078
Rotation	2 (0.7%)	5 (1.6%)	0.454
J-CTO score	1.79 ± 1.07	1.94 ± 1.14	0.094
Post-dilation			
Dissection			
None	116 (40.0%)	96 (30.1%)	0.007
A-B	147 (50.7%)	172 (53.9%)	
C-F	27 (9.3%)	51 (16.0%)	
Treatment Strategy			
DCB-only	147 (50.7%)	/	/
DCB combined with DES	143 (49.3%)	/	/
Dissection after DCB angioplasty			
None	111 (38.3%)	319 (100%)	<0.001
A-B	144 (49.7%)	0	
C-F	35 (12.1%)	0	
Bailout stent	9 (3.1%)	/	/

Values are *n* (%). DCB, drug-coated balloon; DES, drug-eluting stent; LAD, left anterior descending artery; LCX, left circumflex coronary artery; NSE, non-slip element; RCA, right coronary artery.

The mean number of DCB per lesion and length of DCB in the DCB group were 1.41 ± 0.66 and 35.81 ± 19.92 mm, respectively. DES implanted per lesion in the DCB group was significantly less in number (0.75 ± 0.87 vs. 1.99 ± 0.83, *p* < 0.001) and shorter (21.52 ± 25.46 mm vs. 54.45 ± 26.03 mm, *p* < 0.001) than that in the DES-only group. Additionally, the average diameter of DES in the DCB group was smaller (2.82 ± 0.28 mm vs. 2.90 ± 0.35 mm, *p* = 0.018). The total length of DCB + DES in the DCB group was similar with that in the DES-only group (57.33 ± 26.86 mm vs. 54.45 ± 26.03 mm, *p* = 0.180). (Table 3).

**Table 3:** Device characteristics.

	DCB (*n* = 290)	DES-only (*n* = 319)	*p*
Characteristics of DCB			
Number	1.41 ± 0.66	/	/
Length, mm	35.8 ± 19.9	/	/
Diameter, mm	2.63 ± 0.38	/	/
Pressure of inflation, atm	8.03 ± 1.21	/	/
Time of inflation, s	60.9 ± 5.1	/	/
Characteristics of DES			
Number	0.75 ± 0.87	1.99 ± 0.83	<0.001
Length, mm	21.5 ± 25.5	54.5 ± 26.0	<0.001
Diameter, mm	2.82 ± 0.28	2.90 ± 0.35	0.018
Total length of devices	57.3 ± 26.9	54.5 ± 26.0	0.180

Values are mean ± SD. DCB, drug-coated balloon; DES, drug-eluting stent.

### 3.3. QCA measurements

QCA measurements showed that the length of lesions was shorter in DCB group than DES-only group (41.82 ± 19.09 mm vs. 45.67 ± 23.70 mm, *p* = 0.027). Post-procedure MLD (1.83 ± 0.43 mm vs. 2.55 ± 0.40 mm, *p* < 0.001), RVD (2.45 ± 0.43 mm vs. 2.87 ± 0.44 mm, *p* < 0.001) and acute lumen gain (1.83 ± 0.43 mm vs. 2.55 ± 0.40 mm, *p* < 0.001) were smaller in the DCB group. Among the patients with follow-up angiographic results at 1 year, no significant differences in the incidence of restenosis (20.5% vs. 19.7%, *p* > 0.999) or reocclusion (2.7% vs. 2.8%, *p* > 0.999) were observed, and LLL in the DCB group (−0.08 ± 0.65 mm vs. 0.35 ± 0.62 mm, *p* < 0.001) was less than that in the DES-only group (Table 4).

**Table 4:** Quantitative coronary angiography measurements.

	DCB (*n* = 290)	DES-only (*n* = 319)	*p*
Before PCI			
Lesion length, mm	41.8 ± 19.1	45.7 ± 23.7	0.027
Immediately after PCI			
MLD, mm	1.83 ± 0.43	2.55 ± 0.40	<0.001
RVD, mm	2.45 ± 0.43	2.87 ± 0.44	<0.001
Diameter stenosis, %	25.4 ± 9.2	10.9 ± 3.1	<0.001
Acute lumen gain, mm	1.83 ± 0.43	2.55 ± 0.40	<0.001
Angiographic follow-up at 1 year			
No. of patients	112 (38.6%)	71 (22.3%)	<0.001
RVD, mm	2.65 ± 0.51	2.97 ± 0.42	<0.001
MLD, mm	1.86 ± 0.65	2.28 ± 0.63	
Diameter stenosis, %	30.5 ± 18.0	22.8 ± 18.0	0.005
Restenosis	23 (20.5%)	14 (19.7%)	>0.999
Total occlusion	3 (2.7%)	2 (2.8%)	>0.999
Late lumen loss, mm	−0.08 ± 0.65	0.35 ± 0.62	<0.001
Late lumen enlargement	68 (60.7%)	/	/

Values are mean ± SD or *n* (%). DCB, drug-coated balloon; DES, drug-eluting stent; MLD, minimum luminal diameter; PCI, percutaneous coronary intervention; RVD, reference vessel diameter.

### 3.4. Clinical outcomes

**Figure 2:**
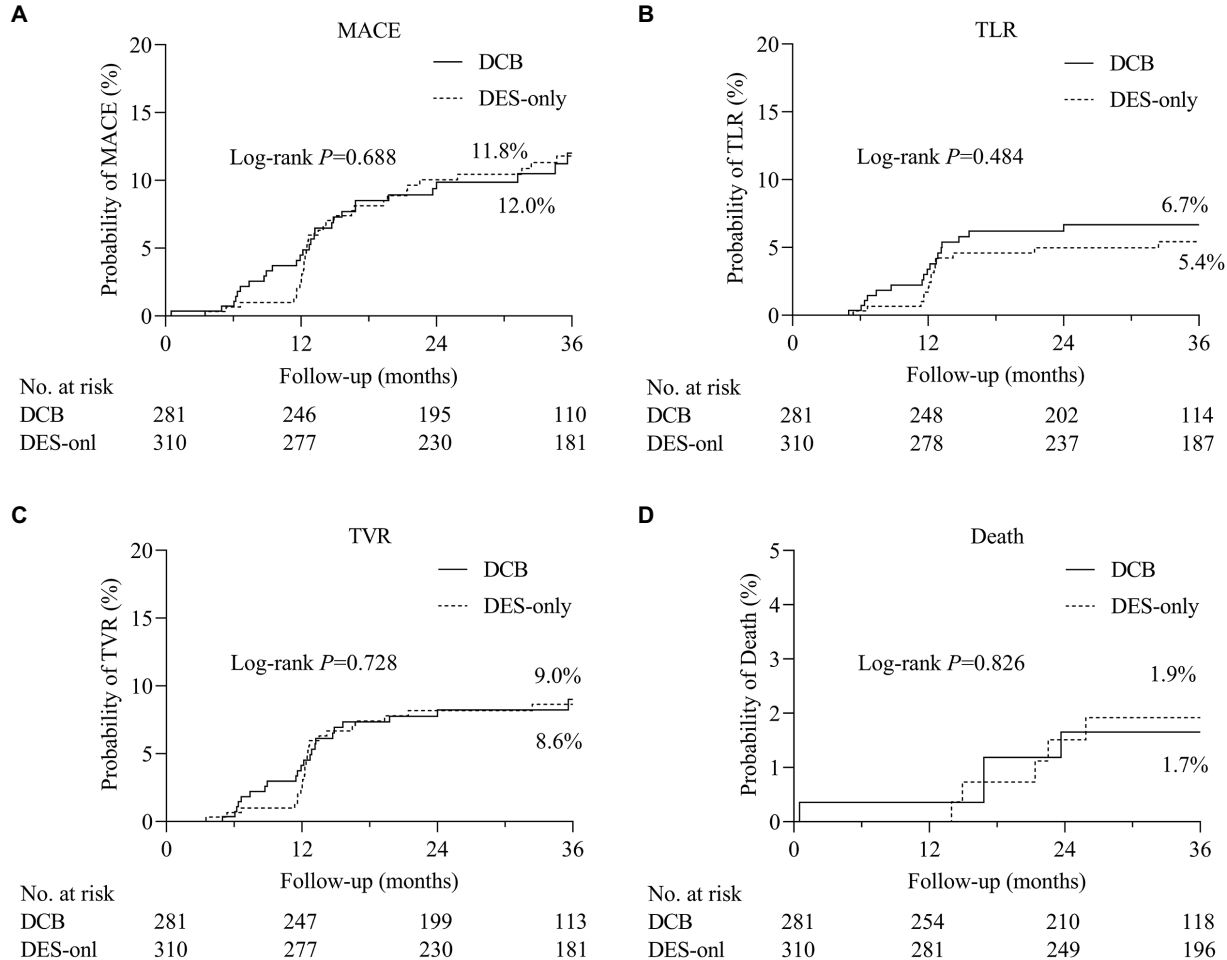
The comparisons of cumulative events between DCB and DES-only group. Kaplan–Meier estimates of the rates of **(A)** major adverse cardiovascular events (MACE), **(B)** target lesion revascularization (TLR), **(C)** target vessel revascularization (TVR), and **(D)** death. DCB, drug-coated balloon; DES, drug-eluting stent.

During hospitalization, no serious adverse events (definite or probable ST, MI, or death) were observed (Table 5). During the 3 years of follow-up, no target lesion thrombosis occurred in any of the groups. Compared with the DES-only group, the Kaplan–Meier estimates of TLR (6.7% vs. 5.4%, log-rank *p* = 0.484) and MACE (12.0% vs. 11.8%, log-rank *p* = 0.688) at 3 years were similar in the DCB group (Figure 2). A multivariate Cox proportional regression model was used to adjust for potential confounders (Table 6). No difference in the risk of MACE was observed between groups (Hazard ration [HR] = 0.932, 95% confidence interval [CI] = 0.532–1.633, *p* = 0.805). Figure 2 shows the Kaplan–Meier survival curves of the endpoints.

**Table 5:** Cumulative clinical events of patients.

	DCB (*n* = 281)	DES-only (*n* = 310)	Log-rank *p*
In-hospital			
ST (definite/probable)	0	0	>0.999
MI	0	0	>0.999
Death	0	0	>0.999
Duration of mean follow-up	35.03 ± 16.83	46.10 ± 22.18	<0.001
Clinical endpoint at 3-year			
TLR	17 (6.0%)	15 (4.8%)	0.484
TVR	23 (8.2%)	24 (7.7%)	0.598
MI	2 (0.7%)	3 (1.0%)	0.943
ST (definite/probable)	0	0	>0.999
All cause death	7 (2.5%)	11 (3.5%)	0.826
Cardiac death	6 (2.1%)	8 (2.6%)	0.703
Non-cardiac death	1 (0.3%)	3 (1.0%)	0.790
MACE^*^	32 (11.4%)	38 (12.3%)	0.688

Values are mean ± SD or *n* (%). ^*^MACE defined as the composite outcome of all-cause death, non-fatal myocardial infarction and target vessel revascularization (including periprocedural). DCB, drug-coated balloon; DES, drug-eluting stent; MACE, major adverse cardiovascular events; MI, myocardial infarction; ST, stent thrombosis; TLR, target lesion revascularization; TVR, target vessel revascularization.

**Table 6:** Multivariate Cox regression analyzes of MACE.

Variables	Hazard ratio (95% CI)	*P*
DCB (vs. DES-only)	1.073 (0.612–1.881)	0.805
Age (≥65 vs. <65)	0.962 (0.573–1.616)	0.885
Male	0.832 (0.455–1.521)	0.551
Number of diseased vessels (vs. 1)		
2	1.283 (0.624–2.642)	0.498
3	1.209 (0.549–2.661)	0.638
Hypertension	0.908 (0.549–1.503)	0.486
Diabetes mellitus	1.188 (0.731–1.930)	0.708
Hypercholesterolemia	0.692 (0.428–1.119)	0.133
Current/ex-smoker	0.820 (0.465–1.447)	0.494
Family history of CAD	1.804 (1.052–3.093)	0.032
Ejection fraction (≥50% vs. <50%)	1.075 (0.525–2.204)	0.843
Target vessel (vs. LAD)		
LCX	1.244 (0.603–2.567)	0.554
RCA	1.331 (0.685–2.587)	0.398
Calcification	0.770 (0.347–1.709)	0.520
Lesion length (≥25 mm vs. <25 mm)	1.189 (0.641–2.204)	0.583
MLD after PCI (≥2.5 mm vs. <2.5 mm)	0.959 (0.492–1.870)	0.903
RVD (≥2.5 mm vs. <2.5 mm)	0.939 (0.531–1.663)	0.830

The *p* values for interaction are derived from Cox proportional hazard models. CAD, coronary artery disease; CI, confidence interval; DCB, drug-coated balloon; DES, drug-eluting stent; LAD, left anterior descending artery; LCX, left circumflex coronary artery; MACE, major adverse cardiovascular events; MLD, minimum luminal diameter; PCI, percutaneous coronary intervention; RVD, reference vessel diameter; RCA, right coronary artery.

## 4. Discussion

To our knowledge, this is the first study to examine the long-term effects of DCB and DES in the treatment of *de novo* CTO lesions. This prospective, multicenter, observational study demonstrated that the less DES strategy (DCB with or without DES) was effective and safe, with no significant differences in the incidence of MACE during long-term follow-up.

CTO, as a challenging obstacle in PCI accounts for 15–25% of elective coronary angiographies (1, 2). With the development of intervention techniques and concepts for recanalization of CTO, the rates of successful recanalization have improved (5). Compared to optimal medical therapy, successful revascularization through PCI can effectively reduce the clinical symptoms related with myocardial ischemia (3, 4). CTO-PCI with complete new-generation DES implantation has satisfactory clinical outcomes. However, concerns about undersized stents after balloon angioplasty, ST, and stent malapposition remain (7, 8).

DCB was originally designed to treat coronary ISR after BMS implantation (9). Clinical trials have demonstrated the efficacy of DCB in BMS/DES-ISR (10). Meanwhile evidence regarding the use of DCB in treating small vessels and bifurcation lesions is accumulating (11). Previous studies have demonstrated that the DCB-only strategy for CTO is a feasible and well-tolerated approach (12, 13). We aimed to investigate the impact of long-term angiographic and clinical outcomes of DCB treatment (DCB alone or combined with DES) in *de novo* CTO lesions.

This study demonstrated encouraging long-term outcomes of DCB-only or combined with DES in CTO-PCI, with low rates of MACE (12.0%). In the DECISION-CTO trial (20), the primary endpoint rate (death, MI, stroke, or any revascularization) in the CTO-PCI group (receiving DES implantation) was 21.5% at 3 years, which was comparable to our results. Furthermore, no target lesion thrombosis was observed during hospitalization or follow up. The low thrombosis rate associated with the target lesion in our study was similar to that reported in a previous study.

In our study, the restenosis (20.5% vs. 19.7%, *p* > 0.999) and reocclusion rates (2.7% vs. 2.8%, *p* > 0.999) at 1-year follow-up between the DCB and DES-only groups were similar. In the DCB-CTO study by Köln et al. (13), the restenosis rate was 11.8% (4/34) and re-occlusion rate was 5.9% with a mean follow-up of 8.6 ± 9.3 months (2/34). In another study on DCB treatment in *de novo* coronary lesions (including bifurcation lesions and CTO lesions) (12), the CTO lesion subgroup had a restenosis rate of 17% (2/12) at a follow-up of 8.2 ± 4.0 months after PCI. Yang et al. (21) conducted a systematic review and meta-analysis comparing the use of BMS and DES in the treatment of coronary CTO lesions. The restenosis rates until 6 months after operation in the DES and re-occlusion groups were 14.21 and 3.95%, respectively. Our study further demonstrated that the patients with CTO lesions receiving less DES strategy have an acceptable restenosis rate in a long-term follow-up period.

One major benefit of DCB therapy for CTO lesions is the possibility of vessel remodeling over time. The LLL until 1-year angiographic follow-up in the DCB group was significantly less than that in the DES-only group (−0.08 ± 0.65 mm vs. 0.35 ± 0.62 mm, *p* < 0.001). One possible reason might be attributed to late lumen enlargement (LLE) occurring in the DCB group. In the DCB group, 60.7% (68/112) had an enlarged MLD at the target vessel lesions on follow-up angiography. Scheller et al. (22) first reported LLE after DCB angiography of *de novo* coronary lesions and then published a study evaluating LLE after DCB angioplasty using QCA analysis (23). The study consecutively included 58 *de novo* lesions that were treated with DCB. The average RVD was 2.58 ± 0.47 mm. After an average follow-up of 4.1 ± 2.1 months, the minimum lumen diameter of the target lesion increased from 1.75 ± 0.55 mm to 1.91 ± 0.55 mm (*p* < 0.001), LLE occurred in 69% of patients. Several subsequent studies (24, 25) have observed the LLE phenomenon after DCB angioplasty for the treatment of *de novo* lesions. LLE associated with DCB angioplasty reduces the incidence of blood-limiting restenosis and has positive implications on patient prognosis as CTO lesions are characterized by negative remodeling. DCB has natural advantages over DES implantation, such as avoid ST risk, allowing the coronary artery to respond to vasomotor reflexes and vessel enlargement without concerns of small-sized DES during PCI and late or very late stent malapposition due to positive remodeling after recanalization.

Meticulous CTO-PCI operations and appropriate lesion preparation are important for reducing stent implantation in CTO revascularization (9). Lesion preparation has been consistently highlighted in DCB angioplasty to achieve optimal results (26), particularly in CTO lesions. Noncompliant, scoring, and cutting balloons were used more frequently in the DCB group to acquire adequate blood flow and sufficient lumen gain. Non-conventional balloons, such as scoring and cutting balloons, can reduce irregular tears at the target lesions and avoid severe coronary dissection when performing DCB angioplasty. Sufficient lesion preparation can increase the contact area between the DCB surface and the intima, and local dissection without flow limitation contributes to the delivery of anti-proliferative drugs, which is a cause of late lumen enlargement during angiographic follow-up. It should be mentioned that bailout stents were not implanted in the lesions after predilation between 30 and 50% stenosis with TIMI flow grade 3, which was not in accordance with the consensus rules (11). The situation is mainly due to interventionists avoiding small-sized stent implantation in distal lesions to reduce the incidence of adverse events in the future.

A hybrid method combining DES and DCB is an acceptable option for the treatment of diffuse coronary diseases (27). DCB as part of a hybrid revascularization technique with DES might be an option for stent length reduction in vessels with CTO lesions (particularly with diffuse disease after CTO restoration). It is well known that a longer stent length is associated with a higher incidence of MACE. Additionally, in the setting of recanalized CTO involving bifurcation or small vessel lesion, DCB provide an intervention option of “leave nothing behind” to avoid jailed ostial lesions or small caged vessel caused by DES. Each CTO is unique and our study provides a flexible and feasible strategy in CTO-PCI based on a combination of DCB and DES to reduce permanent stent length while maintaining the scaffolding properties of stents where they are required. Another potential benefit is the theoretically shorter DAPT duration in CTO lesions treated with DCB-only and a lower risk of bleeding events. Following DCB-only PCI, a 4 week DAPT was considered sufficient, rather than the 6–12 months advised after DES. This is particularly important for those who are at an increased risk of bleeding, as measured by the CRUSADE score, or scheduled for surgery shortly after PCI. Consequently, DCB has the potential to be used as an adjuvant or definitive treatment for CTO.

This study further accumulated clinical experience for DCB in CTO lesions; however, it has the following limitations. First, the current study was a nonrandomized observational study with potential bias, particularly because the intervention strategy was selected by the cardiovascular team. Interventionists may prefer DCB angioplasty for segments of the target lesion with favorable predilation results, and we cannot determine the exact proportion of lesions that cannot be treated with DCB. Second, we did not analyze the changes in ischemic symptoms before and after PCI and compared the improvements between the DCB-and DES-only groups. Furthermore, despite our analysis of restenosis and re-occlusion at 1 year, the population of angiographic follow-up varies, which could influence the results. Larger randomized controlled trials are necessary to evaluate DCB use in *de novo* CTO.

In conclusion, PCI with DCB is a potential “stent-less” therapy in treating *de novo* CTO lesions with satisfactory long-term clinical results compared to the DES-only approach if the result after preparation is satisfactory.

## Data availability statement

The original contributions presented in the study are included in the article/Supplementary material, further inquiries can be directed to the corresponding author.

## Ethics statement

The studies involving human participants were reviewed and approved by First Affiliated Hospital of Zhengzhou University Jincheng People’s Hospital, Jincheng, the Fifth Affiliated Hospital of Zhengzhou University. The patients/participants provided their written informed consent to participate in this study.

## Author contributions

CQ, WL, ZHa, XiW, and XY: conceptualization. XY, SP, XuW, PQ, YS, YZ, PZ, and ZQ: data curation. CQ and XY: formal analysis. CQ, and ZHa: funding and acquisition. CQ, WL, and ZHa: methodology. CQ and ZHu: project administration, resources and supervision. CQ, ZHu, GS, XQ, SP, and PZ: resources. XiW and XY: visualization. XiW and XY: writing original draft. All authors: investigation and writing, review and editing. All authors gave final approval of the manuscript and agreed to be accountable for all aspects of work ensuring integrity and accuracy.

## Conflict of interest

The authors declare that the research was conducted in the absence of any commercial or financial relationships that could be construed as a potential conflict of interest.

## Publisher’s note

All claims expressed in this article are solely those of the authors and do not necessarily represent those of their affiliated organizations, or those of the publisher, the editors and the reviewers. Any product that may be evaluated in this article, or claim that may be made by its manufacturer, is not guaranteed or endorsed by the publisher.
