# Supplementary material for: Long-term outcomes of less drug-eluting stents by the use of drug-coated balloons in de novo coronary chronic total occlusion intervention: A multicenter observational study
Source: Front Cardiovasc Med. 2023 Mar 3;10:1045859. doi: 10.3389/fcvm.2023.1045859 (PMC10022494; doi:10.3389/fcvm.2023.1045859)
Supplement: Supplementary file 1 [file Table_1.DOCX]

Supplementary Material

**Table 1** Characteristics of procedures and lesions.

|  | DCB-only  (n=147) | DES-only  (n=319) | DCB+DES  (n=143) | *P^*^* | *P^**^* |
| --- | --- | --- | --- | --- | --- |
| Access |  |  |  |  |  |
| Trans-radial | 137 (93.2%) | 291 (91.2%) | 133 (93.0%) | 0.585 | 0.689 |
| Trans-femoral | 10 (6.8%) | 28 (8.8%) | 10 (7.0%) |  |  |
| Target vessel |  |  |  |  |  |
| LAD | 48 (32.7%) | 116 (36.4%) | 67 (46.9%) | 0.003 | <0.001 |
| LCX | 40 (27.2%) | 45 (14.1%) | 19 (13.3%) |  |  |
| RCA | 59 (40.1%) | 158 (49.5%) | 57 (39.9%) |  |  |
| Pre-dilation |  |  |  |  |  |
| Semi-compliant balloon (%) | 142 (96.6%) | 311 (97.5%) | 135 (94.4%) | 0.559 | 0.243 |
| Non-compliant balloon (%) | 20 (13.6%) | 17 (5.3%) | 25 (17.5%) | 0.003 | <0.001 |
| Cutting balloon (%) | 29 (19.7%) | 33 (10.3%) | 27 (18.9%) | 0.008 | 0.007 |
| NSE balloon (%) | 38 (25.9%) | 24 (7.5%) | 41(28.7%) | <0.001 | <0.001 |
| Dual wire balloon (%) | 10 (6.8%) | 6 (1.9%) | 3 (2.1%) | 0.011 | 0.013 |
| Moderate/severe Calcification | 5 (3.4%) | 38 (11.9%) | 17 (11.9%) | 0.003 | 0.011 |
| Rotation | 0 | 5 (1.6%) | 2 (1.4%) | 0.332 | 0.320 |
| J-CTO score | 1.83 ± 1.08 | 1.94 ± 1.14 | 1.75 ± 1.08 | 0.324 | 0.209 |
| Post-dilation |  |  |  |  |  |
| Dissection |  |  |  |  |  |
| None | 47 (32.0%) | 96 (30.1%) | 69 (48.2%) | 0.250 | 0.001 |
| A-B | 85 (57.8%) | 172 (53.9%) | 62 (43.4%) |  |  |
| C-F | 15 (10.2%) | 51 (16.0%) | 12 (8.4%) |  |  |
| Dissection after DCB angioplasty |  |  |  |  |  |
| None | 48 (32.6%) | 319 (100%) | 63 (44.1%) | <0.001 | <0.001 |
| A-B | 78 (53.1%) | 0 | 66 (46.1%) |  |  |
| C-F | 21 (14.3%) | 0 | 14 (9.8%) |  |  |
| Bailout stent | 3 (2.0%) | / | 6 (4.2%) | / | / |

Values are n (%). ^*^Comparison of DCB-only and DES-only group; ^**^Comparison of DCB-only、DES-only group and DCB+DES group.

DCB = drug-coated balloon; DES = drug-eluting stent; LAD = left anterior descending artery; LCX = left circumflex coronary artery; NSE = non-slip element; RCA = right coronary artery.

**Table 2** Device characteristics.

|  | DCB-only  (n=147) | DES-only  (n=319) | DCB+DES  (n=143) | *P^*^* | *P^**^* |
| --- | --- | --- | --- | --- | --- |
| Characteristics of DCB |  |  |  |  |  |
| Number | 1.64 ± 0.78 | / | 1.18 ± 0.40 | / | / |
| Length, mm | 42.34 ± 2.69 | / | 29.10 ± 3.74 | / | / |
| Diameter, mm | 2.60 ± 0.40 | / | 2.66 ± 0.35 | / | / |
| Pressure of inflation, atm | 7.87 ± 1.08 | / | 8.19 ± 1.31 | / | / |
| Time of inflation, s | 60.6 ± 4.3 | / | 61.3 ± 5.8 | / | / |
| Characteristics of DES |  |  |  |  |  |
| Number | / | 1.99 ± 0.83 | 1.52 ± 0.60 | / | / |
| Length, mm | / | 54.5 ± 26.0 | 43.64 ± 18.65 | / | / |
| Diameter, mm | / | 2.90 ± 0.35 | 2.82 ± 0.28 | / | / |
| Total length of devices | 42.34 ± 22.69 | 54.5 ± 26.0 | 72.7 ± 21.6 | <0.001 | <0.001 |

Values are mean ± SD. ^*^Comparison of DCB-only and DES-only group; ^**^Comparison of DCB-only、DES-only group and DCB+DES group.

DCB = drug-coated balloon; DES = drug-eluting stent.

**Table 3** Quantitative coronary angiography measurements.

|  | DCB-only  (n=147) | DES-only  (n=319) | DCB+DES  (n=143) | *P^*^* | *P^**^* |
| --- | --- | --- | --- | --- | --- |
| Before PCI |  |  |  |  |  |
| Lesion length, mm | 32.88 ± 16.55 | 45.7 ± 23.7 | 51.35 ± 16.97 | <0.001 | <0.001 |
| Immediately after PCI |  |  |  |  |  |
| MLD, mm | 1.74 ± 0.40 | 2.55 ± 0.40 | 1.93 ± 0.43 | <0.001 | <0.001 |
| RVD, mm | 2.32 ± 0.41 | 2.87 ± 0.44 | 2.57 ± 0.40 | <0.001 | <0.001 |
| Diameter stenosis, % | 25.6 ± 8.3 | 10.9 ± 3.1 | 25.2 ± 10.1 | <0.001 | <0.001 |
| Acute lumen gain, mm | 1.74 ± 0.40 | 2.55 ± 0.40 | 1.93 ± 0.43 | <0.001 | <0.001 |
| Angiographic follow-up at 1 year |  |  |  |  |  |
| No. of patients | 60 (40.8%) | 71 (22.3%) | 52 (36.4%) | <0.001 | <0.001 |
| RVD, mm | 2.55 ± 0.54 | 2.97 ± 0.42 | 2.78 ± 0.45 | <0.001 | <0.001 |
| MLD, mm | 1.86 ± 0.61 | 2.28 ± 0.63 | 1.87 ± 0.69 | <0.001 | <0.001 |
| Diameter stenosis, % | 27.80 ± 15.02 | 22.8 ± 18.0 | 33.71 ± 20.59 | 0.023 | 0.005 |
| Restenosis | 8 (13.3%) | 14 (19.7%) | 15 (28.8%) | 0.359 | 0.124 |
| Total occlusion | 1 (1.7%) | 2 (2.8%) | 2 (3.8%) | >0.999 | 0.778 |
| Late lumen loss, mm | -0.17 ± 0.52 | 0.35 ± 0.62 | 0.04 ± 0.77 | <0.001 | <0.001 |
| Late lumen enlargement | 40 (66.7%) | / | 28 (53.8%) | / | / |

Values are mean ± SD or n (%). ^*^Comparison of DCB-only and DES-only group; ^**^Comparison of DCB-only、DES-only group and DCB+DES group.

DCB = drug-coated balloon; DES = drug-eluting stent; MLD = minimum luminal diameter; PCI = percutaneous coronary intervention; RVD = reference vessel diameter.

**Table 4** Cumulative clinical events of patients.

|  | DCB-only  (n=140) | DES-only  (n=310) | DCB+DES  (n=141) | *P^*^* | *P^**^* |
| --- | --- | --- | --- | --- | --- |
| In-hospital |  |  |  |  |  |
| ST (definite/probable) | 0 | 0 | 0 | >0.999 | >0.999 |
| MI | 0 | 0 | 0 | >0.999 | >0.999 |
| Death | 0 | 0 | 0 | >0.999 | >0.999 |
| Duration of mean follow-up | 34.22 ± 17.81 | 46.10 ± 22.18 | 35.84 ± 15.82 | <0.001 | <0.001 |
| Clinical endpoint at 3-year |  |  |  |  |  |
| TLR | 6 (4.3%) | 15 (4.8%) | 11 (7.8%) | >0.999 | 0.347 |
| TVR | 9 (6.4%) | 24 (7.7%) | 14 (9.9%) | 0.700 | 0.545 |
| MI | 2 (1.4%) | 3 (1.0%) | 0 | 0.649 | 0.402 |
| ST (definite/probable) | 0 | 0 | 0 | >0.999 | >0.999 |
| All cause death | 4 (2.9%) | 11 (3.5%) | 3 (2.1%) | >0.999 | 0.402 |
| Cardiac death | 4 (2.9%) | 8 (2.6%) | 2 (1.4%) | >0.999 | 0.686 |
| Non-cardiac death | 0 | 3 (1.0%) | 1 (0.7%) | 0.556 | 0.510 |
| MACE^✝^ | 15 (10.7%) | 38 (12.3%) | 17 (12.1%) | 0.753 | 0.892 |

Values are mean ± SD or n (%). ^*^Comparison of DCB-only and DES-only group; ^**^Comparison of DCB-only、DES-only group and DCB+DES group. ^✝^MACE defined as the composite outcome of all-cause death, non-fatal myocardial infarction and target vessel revascularization (including periprocedural).

DCB = drug-coated balloon; DES = drug-eluting stent; MACE = major adverse cardiovascular events; MI = myocardial infarction; ST = stent thrombosis; TLR = target lesion revascularization; TVR = target vessel revascularization.
